# Poor Adherence to Mediterranean Diet and Serum Lipopolysaccharide Are Associated with Oxidative Stress in Patients with Non-Alcoholic Fatty Liver Disease

**DOI:** 10.3390/nu12061732

**Published:** 2020-06-10

**Authors:** Francesco Baratta, Daniele Pastori, Simona Bartimoccia, Vittoria Cammisotto, Nicholas Cocomello, Alessandra Colantoni, Cristina Nocella, Roberto Carnevale, Domenico Ferro, Francesco Angelico, Francesco Violi, Maria Del Ben

**Affiliations:** 1 I Clinica Medica, Department of Clinical Internal, Anestesiological and Cardiovascular Sciences, Sapienza University of Rome, 00185 Roma, Italy; francesco.baratta@uniroma1.it (F.B.); daniele.pastori@uniroma1.it (D.P.); simona.bartimoccia@uniroma1.it (S.B.); vittoria.cammisotto@uniroma1.it (V.C.); nicholas.cocomello@gmail.com (N.C.); alessandracolantoni@libero.it (A.C.); cristina.nocella@uniroma1.it (C.N.); domenico.ferro@uniroma1.it (D.F.); francesco.violi@uniroma1.it (F.V.); maria.delben@uniroma1.it (M.D.B.); 2 Department of Medical-Surgical Sciences and Biotechnologies, Sapienza University of Rome, 04015 Latina, Italy; roberto.carnevale@uniroma1.it; 3 Mediterranea Cardio Centro, 80122 Napoli, Italy; 4 Department of Public Health and Infectious Diseases, Sapienza University of Rome, 00185 Roma, Italy

**Keywords:** Non-alcoholic fatty liver disease, Mediterranean diet, oxidative stress, lipopolysaccharide, Nox2-derived peptide

## Abstract

Oxidative stress plays a pivotal role in non-alcoholic fatty liver disease (NAFLD). Factors inducing oxidative stress in NAFLD may be several; however, a relationship with the adherence to Mediterranean Diet (Med-diet) and with serum lipopolysaccharide (LPS) has been poorly investigated in this setting. The aim was to investigate factors associated with impaired oxidative stress in NAFLD, focusing on the potential role of LPS and Med-diet. We enrolled 238 consecutive outpatients from the PLINIO study, in whom we measured the soluble Nox2-derived peptide (sNox2-dp), a marker of systemic oxidative stress, and serum LPS. Adherence to Med-diet was investigated by a nine-item validated dietary questionnaire. Serum sNox2-dp and LPS were higher in patients with NAFLD compared to those without (25.0 vs. 9.0 pg/mL, *p* < 0.001 and 62.0 vs. 44.9 pg/mL, *p* < 0.001, respectively). In patients with NAFLD, the highest sNox2-dp tertile was associated with the top serum LPS tertile (Odds Ratio (OR): 4.71; *p* < 0.001), APRI > 0.7 (OR: 6.96; *p* = 0.005) and Med-diet-score > 6 (OR: 0.14; *p* = 0.026). Analyzing individual foods, the daily consumption of wine (OR: 0.29, *p* = 0.046) and the adequate weekly consumption of fish (OR: 0.32, *p* = 0.030) inversely correlated with the top sNox2-dp tertile. In conclusion, patients with NAFLD showed impaired oxidative stress. Levels of sNox2 correlated with serum LPS and with low adherence to Med-Diet.

## 1. Introduction

Non-alcoholic fatty liver disease (NAFLD) is the most common liver disease worldwide [1]. The spread of NAFLD is strongly associated with the increasing prevalence of obesity and type 2 diabetes because of changing lifestyles and unhealthy dietary habits [2].

Based on the so-called “multiple parallel hits hypothesis”, oxidative stress plays a pivotal role in NAFLD onset and progression from simple steatosis (NAFL) to steatohepatitis (NASH) [3]. The increased production of reactive oxygen species (ROS) is known to cause lipid peroxidation, followed by inflammation, and activation of stellate cells leading to fibrogenesis [4]. Soluble NADPH oxidase 2-derived peptide (sNox2-dp) is a marker of NADPH oxidase activation, which is a major source of extracellular ROS [5]. Elevated serum sNox2-dp levels have been described in several chronic inflammatory and metabolic diseases, including NAFLD [6,7]. In a previous study, we demonstrated increased markers of systemic oxidative stress in subjects with NAFLD. Thus, sNox2-dp levels were increased independently from obesity, diabetes and metabolic syndrome and correlated with the severity of liver steatosis at ultrasound [8].

Factors inducing oxidative stress in NAFLD are still unknown and few studies have addressed the association between oxidative stress and gut microbiota in this setting [9,10,11,12], but there are no evidences on the association between circulating bacterial products and systemic oxidative stress in humans. Clinical and experimental data demonstrated that intestinal bacterial overgrowth and augmented gut permeability are associated with NAFLD and evidence regarding the causal role of gut microbiota in NAFLD is increasing [13]. Serum lipopolysaccharide (LPS) from Escherichia Coli may represent a possible link between dysbiosis and human NAFLD [14,15]. LPS binds to the toll-like receptor 4 (TLR4) complex, triggering a low-grade inflammatory reaction and insulin resistance, two major players in the multifactorial pathogenesis of NAFLD [16]. Recently, we demonstrated an increased LPS localization into liver cells from biopsy proven human and experimental NAFLD, which was significantly associated with liver inflammation through a TLR4 pathway [16].

Infections, leaky gut and dysbiosis, binge drinking and high fructose and saturated fat diet are all possible causes of increased systemic LPS levels. The role of the different components of the diet during the postprandial phase seems to be very important as LPS is absorbed and translocated to the liver for detoxification by APOB-48-containing lipoproteins, such as chylomicrons [17]. Recently, we have demonstrated that gut-derived LPS increases post-prandial oxidative stress via Nox2 activation in patients with impaired fasting glucose [18]. Furthermore, serum levels of LPS have been reported to be related to unhealthy dietary habits and above all with low adherence to Mediterranean style diet (Med-diet) in the general population [19,20,21], but data on NAFLD patients are lacking.

The aim was to investigate the relationship between oxidative stress and serum LPS and if adherence to Med-diet may affect these variables in patients with NAFLD.

## 2. Materials and Methods

The study has been carried out within the ongoing PLINIO study (Progression of Liver Damage and Cardiometabolic Disorders in Non-alcoholic Fatty Liver disease: an Observational Cohort study. ClinicalTrials.gov Identifier: NCT04036357). We enrolled 238 consecutive outpatients referred to the Day Service of Internal Medicine and Metabolic Disorders of the Policlinico Umberto I University Hospital in Rome with at least one out of the following cardio-metabolic diseases: arterial hypertension, overweight/obesity, type 2 diabetes, dyslipidemia, atrial fibrillation (AF), metabolic syndrome (MetS). Exclusion criteria were: average daily consumption of alcohol > 20 g in women and of >30 g in men; excessive drinking and alcohol use were further confirmed by the use of Alcohol Use Disorders Identification Test, AUDIT [22]; presence of hepatitis B surface antigen and antibody to hepatitis C virus; positive tests for autoimmune hepatitis; cirrhosis and other chronic liver diseases; diagnosis of oncological diseases and concomitant therapy with drugs known to promote liver steatosis (e.g., amiodarone).

Patients underwent a complete clinical and biochemical diagnostic work-up including routine clinical and biochemical evaluations. Anthropometric data (i.e., waist circumference and body mass index, BMI) and information on concomitant treatment and co-morbidities were registered. High waist circumference was defined as >84 cm in females and >102 in males. Cardiovascular and metabolic risk factors were defined according to international guidelines. Liver ultrasonography (US) scanning was performed to assess the presence of steatosis. Aspartate aminotransferase-to-platelet ratio index (APRI), a non-invasive marker of advanced fibrosis, was calculated and values of APRI > 0.7 were considered positive for predicting significant hepatic fibrosis [23,24].

Informed written consent was obtained, and the study protocol conformed to the ethical guidelines of the 1975 Declaration of Helsinki as reflected in a priori approval by the local ethical board of Sapienza-University of Rome (Ref. 2277 prot. 873/11). All co-authors had access to the study data and had reviewed and approved the final manuscript.

### 2.1. Liver Ultrasonography 

Liver US scanning was performed to assess the presence of steatosis. All US were performed by the same operator who was blinded to laboratory values using a GE Vivid S6 apparatus equipped with a convex 3.5 MHz probe; fatty liver was defined according to Hamaguchi score [25]. Patients with the US signs of cirrhosis or portal hypertension (nodular liver, ascites, portal flow mean velocity < 14 cm/s, inversion of flow in the portal vein, portosystemic collaterals, portal vein diameter > 13 mm, and decreased or no respiratory variation in splenic and superior mesenteric vein diameter, portal/splenic/superior mesenteric vein thrombosis) were excluded [26,27].

### 2.2. Med-Diet Questionnaire

Adherence to the Med-diet was investigated by a short, nine-item validated and previously described [28] dietary questionnaire, which was administered to each patient at study entry by a trained physician with a direct face-to-face interview, blinded to patients’ characteristics. The questionnaire includes the most important cardio-protective foods and has been shown to correlate inversely with cardiovascular risk both in general population and in patients at high cardiovascular risk [29,30]. It assigns 1 point for: (i) olive oil (≥1 spoon/day, i.e., tablespoon of 10 gr of olive oil); (ii) fruit (≥1 serving/day); (iii) vegetables or salad (≥1 serving/day); (iv) both fruit (≥1 serving/day) and vegetables (≥1 serving/day); (v) legumes (≥2 servings/week); (vi) fish (≥3 servings/week); (vii) wine (≥1 glass/day, ≤20 g for women and ≤30 g for men, as for EASL guidelines on NAFLD/NASH [31]; (viii) meat (<1 serving/day); (ix) [both white bread (<1/day) and rice (<1/week)] or whole-grain bread (>5/week). The Med-Diet score resulting from the questionnaire ranges from 0 to 9 points. Patients with 7–9 points at questionnaire were defined as high adherent to Med-diet.

### 2.3. Serum sNox2-dp

To quantify Nox2 activity, we measured serum levels of soluble sNox2-dp, a marker of Nox2 activation, by ELISA method as previously described [32]. Blood samples were kept for 60 min at 37°C and centrifuged at 300 g; serum was stored at −80°C. Values were expressed as pg/mL; intra- and inter-assay coefficients of variation were 5.2% and 6%, respectively. Due to the absence of a cut-off of sNox2-dp to identify impaired oxidative stress, we arbitrarily categorized sNox2-dp into tertiles as previously described [33].

### 2.4. Serum LPS

Serum LPS levels were measured using a commercial ELISA kit (Cusabio). Standards of LPS, purified from Escherichia coli, and blood samples were plated for 2 h at room temperature onto a microplate pre-coated with the antibody specific for LPS. After incubation, samples were read at 450 nm. Values were expressed as pg/mL; intra- and inter-assay coefficients of variation were 8% and 10%, respectively.

### 2.5. Statistical Analysis

Out of 250 screened subjects, 12 were excluded from the study because of missing data (age, sex, serum LPS, serum sNox2-dp, adherence to Med-diet). In each analysis, patients with one or more missing variable were excluded.

We analyzed median differences of sNox2-dp and of LPS between patients with and without liver steatosis.

Afterwards, we analyzed factors associated with impaired oxidative stress in patients with NAFLD. Distribution of variables was assessed by the Kolmogorov–Smirnov test. Categorical variables were reported as number of cases and percentages; normal and non-normal continuous variables were reported as means ± SD and median and interquartile range, respectively. Comparisons among groups were analyzed by a Student’s *t*-test or ANOVA test, Mann–Whitney or Kruskal–Wallis (for continuous variables) or chi-square test (for categorical variables) when appropriate.

Moreover we carried out a multivariable logistic regression using the highest oxidative stress tertile as dependent variable and age (continuous), female sex, statin use, high adherence to Mediterranean Diet (Med-Diet score > 6), highest LPS tertile (>27 pg/mL), APRI > 0.7, diabetes, arterial hypertension and high waist circumference (>88 cm in females and >102 cm in males) as covariates. To test the association between single food consumption and oxidative stress we performed multivariable logistic analyses testing the association between each single food item and the highest sNox2-dp tertile, after correction for age, female sex, statin use, APRI > 0.7, highest LPS tertile, diabetes, arterial hypertension and high waist circumference.

All analyses were performed with SPSS V.22.0 (SPSS Inc., Chicago, IL, USA). All authors had access to the study data and reviewed and approved the final manuscript

## 3. Results

Out of 238 enrolled subjects, 193 (81.1%) had liver steatosis at US. The mean age was 53.1 ± 12.4 years and 43.3% were women.

**Figure 1:**
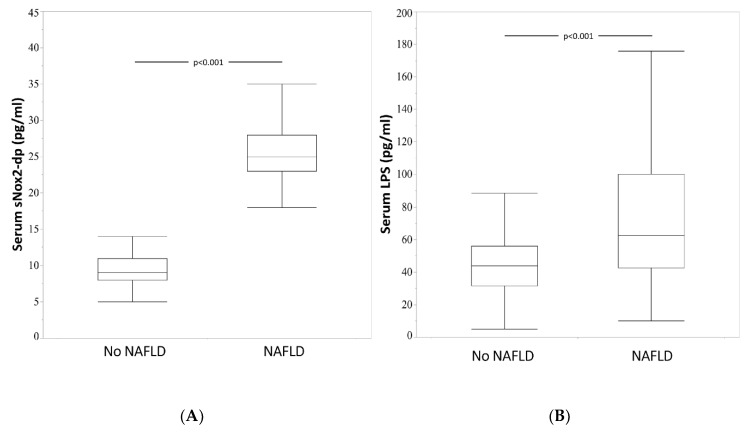
Median values of sNox2 dp (panel **A**) and of serum LPS (panel **B**) in 45 patients without NAFLD and in 193 with patients without NAFLD. sNox2-dp: Soluble NADPH oxidase 2-derived peptide; LPS: lypopolisaccharides; NAFLD: non-alcoholic fatty liver disease.

Serum sNox2-dp (25.0 (23.0–28.0) vs. 9.0 (8.0–11.0) pg/mL, *p* < 0.001) and LPS (62.0 (42.2–99.3) vs. 44.9 (31.8–58.0) pg/mL, *p* < 0.001) were significantly higher in patients with NAFLD in comparison to patients without NAFLD (Figure 1A,B). At bivariate analysis, sNox2-dp correlated with Med-Diet score (rs = −0.138, *p* < 0.029) serum LPS (rs = 0.311, *p* < 0.001) and ALT (rs = 0.484, *p* < 0.001).

### 3.1. NAFLD Patients

Table 1 reports some clinical and biochemical characteristics of NAFLD patients according to serum sNox2-dp tertiles. Patients in the highest sNox2-dp tertile showed higher values of LPS, AST, ALT and GGT and higher prevalence of APRI > 0,7. In patients with APRI ≤ 0.7, the median value of sNox2-dp was 25.0 (23.0–27.7) vs. 35.0(25.0–38.0) pg/mL, *p* = 0.002 in those with APRI > 0.7. 

At multivariable logistic regression analysis, the highest sNox2-dp tertile was directly associated with the highest serum LPS tertile (Odds Ratio (OR): 4.71; *p* < 0.001) and APRI > 0.7 (OR: 6.96; *p* = 0.005), and inversely with the Med-diet score > 6 (good adherence) (OR: 0.14; *p* = 0.026) (Table 2).

### 3.2. Mediterranean Diet

**Figure 2:**
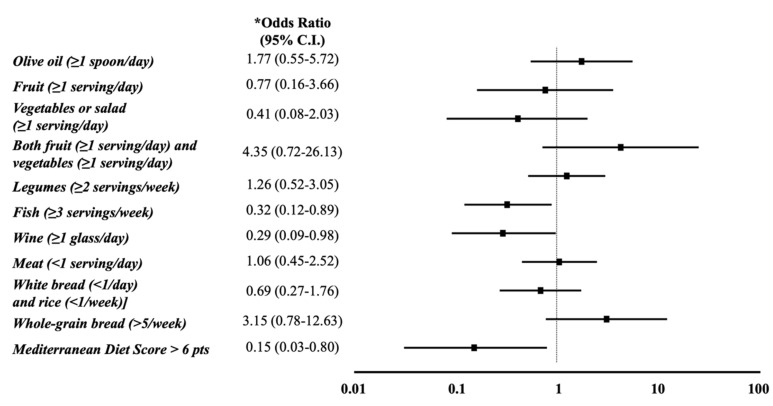
Forest plot of the association of the individual items of the Mediterranean diet questionnaire with the highest sNox2-dp: Soluble NADPH oxidase 2-derived peptide tertile. * After correction for age, sex, statin use, AST-to-platelet ratio index (APRI)>0.7, highest lipopolysaccharide tertile (>27 pg/mL), diabetes, arterial hypertension, high waist circumference.

When we analyzed the association between sNox2-dp and the individual Med-diet questionnaire items, at multivariable logistic regression analysis the daily consumption of wine (OR: 0.29, *p* = 0.046) and the intake of at least two fish servings per week (OR: 0.32, 95% CI: 0.117–0.894, *p* = 0.030) inversely correlated with the top sNox2-dp tertile (Figure 2).

## 4. Discussion

We found increased systemic oxidative stress in NAFLD patients as compared to patients without NAFLD. This result confirms our previous ones showing increased levels of urinary 8-iso-PGF2α and of serum sNox2-dp, together with lower serum vitamin E levels [34], in a large cohort of consecutive NAFLD patients [5]. These findings are also in keeping with recent experimental data suggesting a key role for Nox proteins in the progression of hepatic fibrosis [35] and for Nox2 in the modulation of inflammation in NAFLD [36]. In addition, we found increased level of serum LPS in NAFLD. To note, median serum LPS levels in these patients were higher than those previously measured by ourselves in other clinical settings, such as pre-diabetes [37], atrial fibrillation [38] and cirrhosis [39].

In patients with NAFLD, high level of oxidative stress was independently associated with higher values of LPS and a low adherence to Med-diet. However, from our data we cannot assess whether the increased intestinal LPS translocation in NAFLD is due to a leak of the gut barrier, as we proved in other studies [16,37], or to other translocation mechanisms, as via lipid absorption [17].

Overall, these findings support the hypothesis of a possible role of diet and LPS as stimulus for oxidative stress activation in human NAFLD. The association between oxidative stress and Mediterranean diet was previously described in other settings, such as atrial fibrillation [29], and in Korean and Greek general population [40,41]. There are also some interventional data demonstrating the LPS reduction with a Med-diet enriched with different nutrients [42,43]. However, another possible mechanism accountable for the correlation between low adherence to Med-Diet and oxidative stress could be the previously reported strong association between poor adherence to this dietary model and insulin resistance in NAFLD patients [44]. However, in this study, we did not find any association between metabolic syndrome and oxidative stress [45].

In addition, our study demonstrated a strong association of oxidative stress with APRI, a non-invasive marker of liver fibrosis. This result agrees with our previous study demonstrating the association between systemic oxidative stress and some severity indices of fibrosis in liver biopsies of NAFLD patients [4].

When we analyzed individual food intakes, we found that fish and moderate wine consumption appeared to play a protective role against oxidative stress. The data on wine consumption is consistent with previous works showing a beneficial effect of wine-derived polyphenol, specially resveratrol, on the redox balance [46]. Most data on the association between fish consumption and oxidative stress are derived from studies testing the effect of fish oil as dietary supplementation. While there are wide evidences that the use of fish oil redistributes lipoprotein subclasses without increasing oxidative stress [47], the ability of fish oil to ameliorate redox status is still debated. Conversely, two randomized clinical trials demonstrated that fish oil was not effective in reducing oxidative stress in patients with atrial fibrillation [48] nor in healthy subjects [49]. Notably, in a non-controlled dietary trial, during weight loss, fish intake correlated with oxidative stress reduction better than fish oil supplementation [50]. Finally, despite the fact that the questionnaire we used was not designed to investigate single food intakes, our data show that the whole Mediterranean pattern has a higher effect on sNox2-dp levels compared to the separate effects of wine and fish intakes.

The study may have implications. Serum LPS may represent a novel pathway triggering oxidative stress in NASH. This could suggest dietary and/or therapeutic approaches aimed at reducing gut pathogenic bacterial translocation. However, we believe that the study should be extended to the evaluation of the effects of LPS on oxidative status as a whole and, above all, on antioxidant status.

The study has also some limitations. First, we detected fatty liver by ultrasound, which is a qualitative method inadequate to quantify less than 30% liver fat content. The gold standard for the diagnosis of NAFLD is liver biopsy, but this is an invasive procedure with potentially serious complications and is therefore not acceptable without clinical indication. Second, the study has been carried out in patients recruited in a Hospital-based setting and the protocol design did not contemplate healthy controls.

Finally, the observational design of the study does not make it possible to establish a cause–effect relationship.

## 5. Conclusions

Overall, our results suggest that Med-Diet may be a beneficial nutritional approach in NAFLD patients to improve redox status, but interventional studies are needed to confirm our hypothesis.

## Figures and Tables

**Table 1:** Clinical and biochemical characteristics of NAFLD patients according to sNox2-dp tertiles.

	sNox2-dp Tertiles				
	I (*n* = 72)	II (*n* = 67)	III (*n* = 54)	*p* ^#^	*p*
Age (years)	53.7 ± 12.0	55.4 ± 10.9	48.9 ± 12.2	0.008	0.026 ° 0.395 ^§^ 0.002 ^†^
Women	*n* = 29, 40.3%	*n* = 26, 38.8%	*n* = 26, 48.1%	0.547	0.378 ° 0.859 ^§^ 0.302 ^†^
Smokers	*n* = 17, 23.6%	*n* = 20, 29.9%	*n* = 14, 25.9%	0.703	0.765 ° 0.406 ^§^ 0.633 ^†^
BMI (kg/m^2^) **	31.2 ± 5.4	30.3 ± 4.1	29.4 ± 4.2	0.098	0.042 ° 0.273 ^§^ 0.235 ^†^
Waist circumference (cm) *	107.5 (101.0–116.0)	106.0 (98.7–113.2)	103.0 (97.0–108.5)	0.072	0.027 ° 0.417 ^§^ 0.114 ^†^
Diabetes	*n* = 28, 38.9%	*n* = 17, 25.4%	*n* = 16, 29.6%	0.216	0.281 ° 0.089 ^§^ 0.601 ^†^
Fasting blood glucose (mg/dL) *	113.2 ± 45.1	105.1 ± 26.8	101.0 ± 30.9	0.146	0.090 ° 0.340 ^§^ 0.179 ^†^
Statin use *****	*n* = 37, 52.9%	*n* = 21, 32.3%	*n* = 12, 22.6%	0.002	0.001 ° 0.016 ^§^ 0.245 ^†^
Arterial Hypertension	*n* = 47, 65.3%	*n* = 45, 67.2%	*n* = 24, 44.4%	0.021	0.020 ° 0.814 ^§^ 0.012 ^†^
GGT (UI/L) ****	23.0 (15.0–36.0)	28.0 (18.0–42.5)	39.0 (27.5–109.5)	<0.001	<0.001 ° 0.092 ^§^ 0.001 ^†^
AST (UI/L)	21.0 (17.0–24.0)	20.0 (16.0–30.0)	34.5 (25.0–49.5)	<0.001	<0.001 ° 0.853 ^§^ <0.001 ^†^
ALT (UI/L)	25.0 (19.3–35.8)	24.0 (17.0–36.0)	50.0 (33.8–91.8)	<0.001	<0.001 ° 0.706 ^§^ <0.001 ^†^
Platelets (n/mL)	248.0 (197.0– 283.0)	227.0 (186.0–278.0)	235.0 (197.3–280.0)	0.287	0.499 ° 0.110 ^§^ 0.451 ^†^
APRI ≥ 0.7	*n* = 2, 2.8%	*n* = 3, 4.5%	*n* = 10, 18.5%	0.002	0.004 ° 0.591 ^§^ 0.013 ^†^
LPS (pg/mL)	50.6 (41.4–81.2)	52.0 (39.3–90.0)	85.3 (49.8–136.3)	0.002	0.002 ° 0.683 ^§^ 0.002 ^†^

* data missing in 1 patient, ** data missing in 2 patients, **** data missing in 4 patients, ***** data missing 5 in patients. # *p* among groups; Anova–test, Kruskal–Wallis and chi-square test when applicable. pairwise *p*: ° I vs III tertile, ^§^ I vs II tertile, ^†^ II vs III tertile; t-Student, Mann–Whitney and chi-square test when applicable. ^#^ ≤23 pg/mL; ^##^ >23 and ≤27 pg/mL; ^###^ >27 pg/mL. sNox2-dp: Soluble NADPH oxidase 2-derived peptide; BMI: body mass index; GGT: gamma glutamiltranspeptidase; AST: aspartate transaminase; ALT: alanine transaminase; APRI: AST-to-platelet ratio index; LPS: lipopolysaccharide.

**Table 2:** Multivariable logistic regression analysis of factors associated with the top *sNox2-dp: Soluble NADPH oxidase 2-derived peptide* tertile in 186 patients with NAFLD.

	*p*	Odds Ratio	95% C.I. for Odds Ratio Lower-Upper	
Age (continuous)	0.227	0.98	0.94	1.01
Women	0.973	1.01	0.45	2.29
Statin use	0.062	0.41	0.16	1.05
Smokers	0.608	0.79	0.32	1.94
Good Adherence to Mediterranean Diet (Med-Diet score > 6)	0.026	0.14	0.03	0.79
Highest LPS tertile (>27 pg/mL)	<0.001	4.71	2.11	10.51
APRI>0.7	0.005	6.96	1.80	26.98
Diabetes	0.215	1.81	0.71	4.63
Arterial Hypertension	0.114	0.51	0.22	1.17
High Waist Circumference *	0.220	1.91	0.68	5.37

* >88 cm in females and >102 cm in males; APRI: AST-to-platelet ratio index; LPS: lipopolysaccharide.
